# Comparisons of Allergenic and Metazoan Parasite Proteins: Allergy the Price of Immunity

**DOI:** 10.1371/journal.pcbi.1004546

**Published:** 2015-10-29

**Authors:** Nidhi Tyagi, Edward J Farnell, Colin M Fitzsimmons, Stephanie Ryan, Edridah Tukahebwa, Rick M Maizels, David W Dunne, Janet M Thornton, Nicholas Furnham

**Affiliations:** 1 The EMBL-European Bioinformatics Institute, Wellcome Trust Genome Campus, Hinxton, Cambridge, United Kingdom; 2 Department of Pathology, University of Cambridge, Cambridge, United Kingdom; 3 Institute of Immunology and Infection Research, University of Edinburgh, Edinburgh, United Kingdom; 4 Vector Control Division, Ministry of Health, Kampala, Uganda; 5 London School of Hygiene and Tropical Medicine, London, United Kingdom; Emory University, UNITED STATES

## Abstract

Allergic reactions can be considered as maladaptive IgE immune responses towards environmental antigens. Intriguingly, these mechanisms are observed to be very similar to those implicated in the acquisition of an important degree of immunity against metazoan parasites (helminths and arthropods) in mammalian hosts. Based on the hypothesis that IgE-mediated immune responses evolved in mammals to provide extra protection against metazoan parasites rather than to cause allergy, we predict that the environmental allergens will share key properties with the metazoan parasite antigens that are specifically targeted by IgE in infected human populations. We seek to test this prediction by examining if significant similarity exists between molecular features of allergens and helminth proteins that induce an IgE response in the human host. By employing various computational approaches, 2712 unique protein molecules that are known IgE antigens were searched against a dataset of proteins from helminths and parasitic arthropods, resulting in a comprehensive list of 2445 parasite proteins that show significant similarity through sequence and structure with allergenic proteins. Nearly half of these parasite proteins from 31 species fall within the 10 most abundant allergenic protein domain families (EF-hand, Tropomyosin, CAP, Profilin, Lipocalin, Trypsin-like serine protease, Cupin, BetV1, Expansin and Prolamin). We identified epitopic-like regions in 206 parasite proteins and present the first example of a plant protein (BetV1) that is the commonest allergen in pollen in a worm, and confirming it as the target of IgE in schistosomiasis infected humans. The identification of significant similarity, inclusive of the epitopic regions, between allergens and helminth proteins against which IgE is an observed marker of protective immunity explains the ‘off-target’ effects of the IgE-mediated immune system in allergy. All these findings can impact the discovery and design of molecules used in immunotherapy of allergic conditions.

## Author Summary

Allergy is an increasingly widespread clinical problem that leads to various conditions such as allergic asthma and susceptibility to anaphylactic shock. These conditions arise from exposure to a range of environmental and food proteins (‘allergens’) that are recognised by a form of immune system antibody called IgE. This part of the immune system is thought to have evolved to provide mammals with additional rapid response mechanisms to combat metazoan parasites. Here, we address the pertinent question, ‘what makes an Allergen an Allergen’ as, although they constitute a very small percentage of known proteins, they appear to be diverse and unrelated. Using computational studies, we have established molecular similarity between parasite proteins and allergens that affect the nature of immune response and are able to predict the regions of parasite proteins that potentially share similarity with the IgE-binding region(s) of the allergens. Our experimental studies support the computational predictions, and we can present the first confirmed example of a plant pollen-like protein in a worm that is targeted by IgE. The results of this study will enable us to predict likely allergens in food and environmental organisms and to help design protein molecules to treat allergy in the future.

## Introduction

Allergy is a hypersensitive immune reaction to environmental antigens from diverse sources such as foods, plants and innocuous organisms. The mechanism responsible for eliciting the allergic reaction involves components of the immune system, in particular the IgE antibody isotype, which also mediate the immune response against helminthic infection. Several significant studies have elucidated the mechanisms involved in the immune response to helminth infection and to allergen exposure, and have been comprehensively reviewed in the literature [1–3].

Extensive studies have correlated high levels of parasite-specific IgE antibody in the host with acquired immunity against both helminth endoparasites such as Platyhelminthes (*Schistosoma and Echinococcus*) [4–8] and nematodes (hookworms, *Trichuris* and *Ascaris*) [9–11] as well as arthropod ectoparasites (tick, mites and insects) [12–14]. Also, IgE cross reactivity has been well established between some allergenic proteins and certain metazoan parasite proteins [15–18]. These immunological assays further suggest that not only are similar immune system components involved in acquiring immunity against helminths and in allergic conditions, but that the molecular targets for these responses may also share key characteristics.

Allergenic proteins from a wide variety of sources have been collated and documented in the Allergome database [19] and classified into protein domain families in the Allfam database [20]. The domain families that are populated predominantly by allergenic proteins, represent only around 2% of all protein domain families defined by Pfam [21]. Moreover just 10 protein domain families are reported to represent nearly half of all documented allergenic proteins. Although it has been previously proposed that allergenicity is associated with protease activity [22] and with toxic properties [23], 9 of these 10 fall into neither category. Host IgE responses against several *S*. *mansoni* allergen-like proteins have been previously studied including members of the Tegumental-Allergen-Like (TAL), Tropomysosin and Venom Allergen-Like (VAL) protein domain families [24–27]. During natural infection, antibody responses to *S*. *mansoni* antigens, including those to members of the TAL and Tropomyosin families have been found to depend on the expression patterns throughout the adult worm and eggs. Constant exposure to antigens may result in the induction of a regulated response against excessive IgE including the decreased production of IgE and increased production of anti-inflammatory IgG4 [27–29].

Such a switch in chronic helminth infections are indicated by a modified T-helper 2 (Th2) cell environment, which is characterized by increased T-regulatory cell levels and a predominant IgG4 antibody profile [30]. Contrary to this, unregulated inflammatory responses are characterized by a hyper-responsive immune system, with higher levels of Th1 and reduced T-regulatory cell numbers accompanied by significantly high IgE levels [2]. However, hypo-responsiveness of the immune system can occur in cases of chronic helminth infections and this has been reported to be beneficial in reducing the inflammatory response caused by further infection of certain bacteria and eukaryotic parasites [31,32]. Indeed, infections of *Schistosoma mansoni* and *haematobium* have been observed to alleviate the symptoms of allergy [33,34]. These studies highlight the inverse relationship between helminth infection and atopy when tested against house dust mites and certain aeroallergens.

Based on these observations, Fitzsimmons and Dunne [21] hypothesized that similarity between anti-metazoan parasite and allergic responses may be mirrored in the molecular similarity between allergenic proteins and proteins encoded in genomes of ecto- and endo-parasitic metazoans. Highly specialized immune system components have evolved to combat the effect of infecting metazoan parasites and provide immunity against the infection; however, in the absence of infection, with its attendant immunoregulation (in atopic individuals), this system can switch to the collateral damaging mode and becomes hyper-responsive towards innocuous environmental proteins, possibly due to similar molecular features of the two. Noteworthy, but scarce, examples of studies establishing structure based homology between parasite and allergenic proteins (e.g. dust mite group II allergen, Der p 2 and Der f 2 sharing structure based homology with carbohydrate-binding module of grass allergen expansin proteins) provide modest support to our hypothesis [35].

In the quest to strengthen the hypothesis that the IgE-target proteins in metazoan parasites and allergenic molecules share similar molecular features, the systematic exercise of comparing allergenic and parasite proteins and assessing the epitopic regions of allergenic proteins and ‘epitope-like’ regions of parasite proteins becomes of primary importance.

Here we present a workflow involving computational analyses supported by experimental verification for detecting putative IgE inducing structure/sequence motifs in proteins encoded in genomes of parasites that share molecular similarity with epitopic regions of members of protein families that are populated predominantly by allergenic proteins.

## Materials and Methods

### Generation of datasets

Two main datasets were generated:

#### Dataset 1 (Allergenic proteins, IgE/IgG4-binding peptides)

(a) 2712 unique full-length protein sequences from various sources that are known to cause allergy and/or bind IgE were collated from the Allergome database [19]; (b) 2577 specific fragments from 190 proteins that are known to bind IgE and IgG4 antibodies in immunological assays were retrieved from the Immune Epitope Database (IEDB) database [36].

#### Dataset 2 (Parasite proteins)

Protein sequences encoded in genomes of metazoan parasitic organisms were procured from the UniProt database (January, 2014 release) [37]. Various parasitic organisms belonging to different taxonomic categories that are considered for the analysis are (i) platyhelminthes: *Echinococcus granulosus*, *Fasciola hepatica*, *Schistosoma haematobium*, *Schistosoma japonicum*, *Schistosoma mansoni and Taenia* sp. and (ii) nematodes: *Ascaris lumbricoides*, *Anisakis simplex*, *Brugia malayi*, *Mansonella perstans*, *Nippostrongylus brasiliensis*, *Onchocerca volvulus*, *Trichinella spiralis* and *Trichuris trichiura* (iii) arthropods (mites): *Acarus siro*, *Aleuroglyphus ovatus*, *Blomia tropicalis*, *Dermatophagoides farinae*, *Dermatophagoides microceras*, *Dermatophagoides pteronyssinus*, *Euroglyphus maynei*, *Glycyphagus domesticus*, *Lepidoglyphus destructor*, *Ornithonyssus sylviarum*, *Psoroptes ovis*, *Sarcoptes scabiei* and *Tyrophagus putrescentiae*. A total of 70403 protein sequences encoded in genomes of above-mentioned endo- and ecto-parasites constitute dataset 2.

### Protein domain definition for allergenic proteins

Though the Allfam database provides Pfam domain definitions for allergenic proteins documented in the Allergome database, it has not been updated since 2011. To assign the latest Pfam domain definitions and to include the most recent additions to the Allergome database, we have derived protein domain definitions for dataset 1 and dataset 2 from the Pfam database 27.0. Of these, 10 protein domain families/superfamilies that are reported to represent nearly 45% of all documented allergenic molecules and thus are highly populated with allergenic protein members are considered for further analysis [21]. These protein domain families/superfamilies with their brief description are shown in Table 1.

**Table 1:** List of the protein domain families/superfamilies that are populated predominantly with allergenic molecules. Total number of allergenic molecules (retrieved form Allergome database) in these families/superfamily and their ‘close homologs’ in eukaryotic metazoan parasites are shown.

Sr No.	Pfam domain family/superfamily	Pfam accession	Brief description	No. of allergenic molecules	No. of parasitic proteins
1	EF hand	PF00036, PF01023, PF12763, PF13202, PF13405, PF13499 and PF13833	Calcium binding proteins with helix-loop-helix structural motif	150	362
2	Tropomyosin	PF00261 and PF12718	Coiled-coil cytoskeletal protein	227	81
3	CAP	PF00188	Cysteine-rich secretory protein Antigen 5, and Pathogenesis-related 1 proteins	35	113
4	Profilin	PF00235	Actin-binding proteins	117	13
5	Lipocalin	PF0006, PF08212 and PF02098	Transporters for small hydrophobic molecules such as lipids and steroid hormones	83	42
6	Trypsin-like serine protease	PF00089, PF00431 PF02983, PF09396 and PF12032	Serine protease	46	260
7	Cupin	PF00190 and PF04702	Family comprising of proteins with conserved barrel domain including plant seed storage proteins.	72	130
8	Bet v 1	PF00407	Family of plant allergens	145	58
9	Expansin (Pollen_allerg_1 and DPBB_1)	PF01357 and PF03330	Pollen allergen and Rare lipoprotein A family	57	-
10	Prolamin (Tryp_alpha_amyl)	PF00234	Plant storage proteins	132	-

### Detection of homologs of allergenic proteins in parasites

Homologs of allergenic proteins (dataset 1a) have been detected in parasite proteins (dataset 2) by searching Hidden Markov Models (HMM) profiles of the above mentioned 10 Pfam protein domain families/superfamilies using HMMER3 [38]. We searched parasite proteins that are categorized in the same superfamily or same fold as allergenic proteins according to the CATH (Class, Architecture, Topology, Homology) [39] classification scheme in Gene3D database [40] and collated them.

Gene3D assigns domains to regions of protein sequences with no known 3D structure based on the CATH classification scheme using more sensitive structure-based HMMs derived from a superfamily. Parasite proteins that did not show an association with any CATH homologous superfamily were searched in the Gene3D database to ascertain if they shared the same topology/fold as an allergenic protein to establish similarity/homologous relationships.

Parasite proteins are considered to be a close homolog of the allergenic proteins if both the proteins belong to same family (defined by Pfam) or same homologous superfamily (defined by CATH/Gene3D). However, parasite proteins are regarded as distant homologs if they do not share the same family/superfamily but share a common structure fold with an allergen protein.

To establish the relationship of parasite proteins with allergenic proteins, we culled 3D coordinates of experimentally known structures (if existing in the Protein Data Bank (PDB)) or from homology based models of both proteins (from Modbase [41]) and superimposed corresponding structures using least square superimposition program LSQMAN (http://xray.bmc.uu.se/usf/lsqman_man.html). Homology-based structural models that share more than 30% sequence identity with the template sequence (from any source) are considered for the analysis. The global structural similarity between two proteins was measured using TM (template-modeling) score. A TM score of ≥0.5 associated with structure alignment of a pair of proteins is considered to be statistically significant and the two proteins in the alignment are likely to share the same fold [42].

### Detection of epitope-like fragments in parasite proteins

1. Sequence-based approach: We searched epitopic fragments from allergenic proteins (dataset 1b) in parasitic ‘close homologous’ protein sequences to identify putative IgE/IgG4-binding epitopic-like-regions by employing the FASTA program [43] using default parameters.
2. Structure-based approach: We culled structure motifs, using 3D coordinates for the known structures and homology-based models from PDB and Modbase databases respectively, of epitopic region of allergenic proteins and searched against the structure dataset of parasite proteins using a novel algorithm implemented in the BC Search (Binet-Cauchy) program [44]. The BC search algorithm was developed to provide a structure similarity scoring function for small protein fragments when searched in a large protein structure database, and is therefore suitable for the comparison of small protein fragments such as epitopes.

To observe whether these significantly similar epitopic and epitopic-like regions occur on equivalent positions on respective homologous proteins, a comprehensive analysis was performed by manual inspection/visualization comparing their topology on known 3D structure/models of allergenic and parasite proteins, respectively.

**Fig 1:**
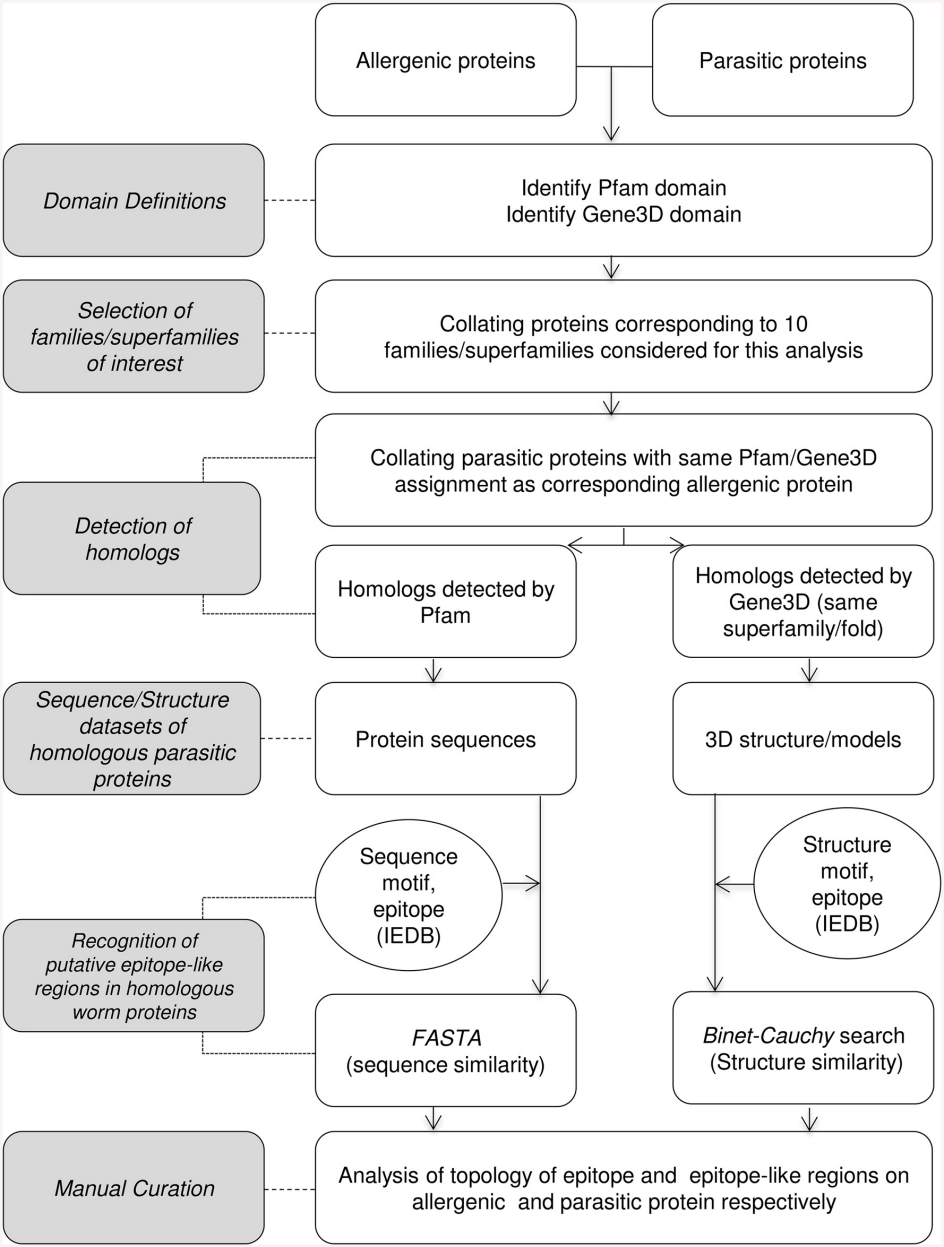
Flowchart depicting the workflow involved in the present analysis.

A summary of the various steps described in the material and methods section is presented in Fig 1.

### Cloning, expression and purification of Bet v 1-like protein from *S*. *mansoni* (SmBv1L)

A Puerto Rican strain of *S*. *mansoni* was used in this study. Total RNA was isolated from adult worms and its integrity was verified using a Bioanalyser (Agilent Technologies, Bracknell, UK) as previously described [45]. cDNA was prepared from 1μg total RNA using random hexamers (Sigma-Aldrich, Gillingham, UK) and Superscript II reverse transcriptase according to the manufacturer’s instructions (Life Technologies, Paisley, UK).

SmBv1L was cloned into the pET15b vector (Merck, UK). PCR to generate a gene-specific coding sequence was performed using Phusion High Fidelity DNA polymerase (Thermo Fisher Scientific, Reading, UK) using the following primers, 5’- TAGGATCCTATGAATGCATATATTATTCG and 5’-ATGGATCCTTATCTAGAGTCGGA at an annealing temperature of 62°C. The PCR product and plasmid were digested with FastDigest BamHI (Thermo Fisher Scientific) and plasmids were treated with FastAP (Thermo Fisher Scientific). Ligation was performed using T4 DNA ligase (Thermo Fisher Scientific) and plasmids transformed into chemically competent DH5alpha *E*. *coli* cells by heat shock. Sanger Sequencing was used to confirm the CDS sequence that was uploaded to GenBank (Accession No: KM281668).

Recombinant SmBv1L was produced by isolation from inclusion bodies and on-column refolding using an AKTAPrime+ according to the manufacturer’s instructions (GE Healthcare). Briefly, overnight cultures of TG2 *E*. *coli* were diluted 1:10, expanded to OD 0.4–0.8 and induced for 3 hours with 1mM IPTG. Pelleted cells were lysed using a French Pressure Cell at ≈10,000 psi and the pellet containing inclusion bodies were isolated by centrifugation at 15,000 x g for 1 hour. Pellets were washed 3 times in cold isolation buffer (2 M urea, 20 mM Tris-HCl, 0.5 M NaCl, 2% Triton-X 100, pH 8.0) and then were incubated in binding buffer (6 M guanidine hydrochloride, 20 mM Tris-HCl, 0.5 M NaCl, 5 mM imidazole, 20 mM β-mercaptoethanol, pH 8.0) overnight at 4°C. Purification was performed by on-column refolding on a HisTrap FF 1 ml column (GE Healthcare), after equilibration of the column and sample binding, the column was washed with solubilisation buffer (6 M urea, 20 mM Tris-HCl, 0.5 M NaCl, 5 mM imidazole, 1 mM β-mercaptoethanol, pH 8.0) and the bound protein refolded in a gradient of refolding buffer (20 mM Tris-HCl, 0.5 M NaCl, 5 mM imidazole, 20 mM β-mercaptoethanol, pH 8.0) and solubilisation buffer. Protein was eluted from the column in a gradient of elution buffer (20 mM Tris-HCl, 0.5 M NaCl, 0.5 M imidazole, 20 mM β-mercaptoethanol, pH 8.0) and collected in fractions 7–20. Refolded SmBv1L was concentrated and buffer exchanged to resuspension buffer (20 mM Tris-HCl, pH 8.0), attached to 1 mL washed HisTrap FF and protein eluted by incubation with 150 U/mg protein Thrombin (GE Healthcare). Thrombin was removed by incubation with benzamadine-agarose beads (Sigma-Aldrich) and the protein buffer exchanged to PBS. Proteins were tested for bacterial contamination by in-house ELISA assay as previously described [24].

### Study population

Venous plasma samples were collected in the fishing village of Namoni on the shores of Lake Victoria, Mayuge District, Uganda, an area of high year-round schistosomiasis (*S*. *mansoni*) transmission occurred. The 372 plasma donors (6–40 years, 57% female, mean age 18) were randomly selected from community members who had detectable *S*. *mansoni* eggs in a single stool sample (microscopically examined in two Kato-Katz thick smears). Blood was collected in heparinized tubes at two time-points, immediately before anti-schistosomiasis treatment with praziquantel (PZQ, 40mg/kg body mass) and 5 weeks post-treatment. Plasma separated by centrifugation (2000 x g for 5 min) was stored at -80°C until use. This analysis focuses on 222 individuals with full parasitological and serological information available both pre- and post-treatment (age 6–40 years, mean age 16 years, 59% female).

### Enzyme-linked immunosorbent assay (ELISA)

Antigen-specific IgG1, IgG4 and IgE levels were measured in the plasma from infected individuals in duplicate by ELISA as detailed previously [24] with the following modifications. SmBv1L was applied to plates in sodium bicarbonate coating buffer at 4°C overnight at a concentration of 0.875 μg/ml. Human plasma was diluted 1:40 for IgE assays and 1:100 for IgG isotypes.

### Data handling and statistics

Data handling and statistical analysis was performed using STATA for Mac version 12.1. Area proportional Venn diagrams were drawn using eulerAPE version 1.0 [46].

## Results

### Distribution of known allergens across taxa

**Fig 2:**
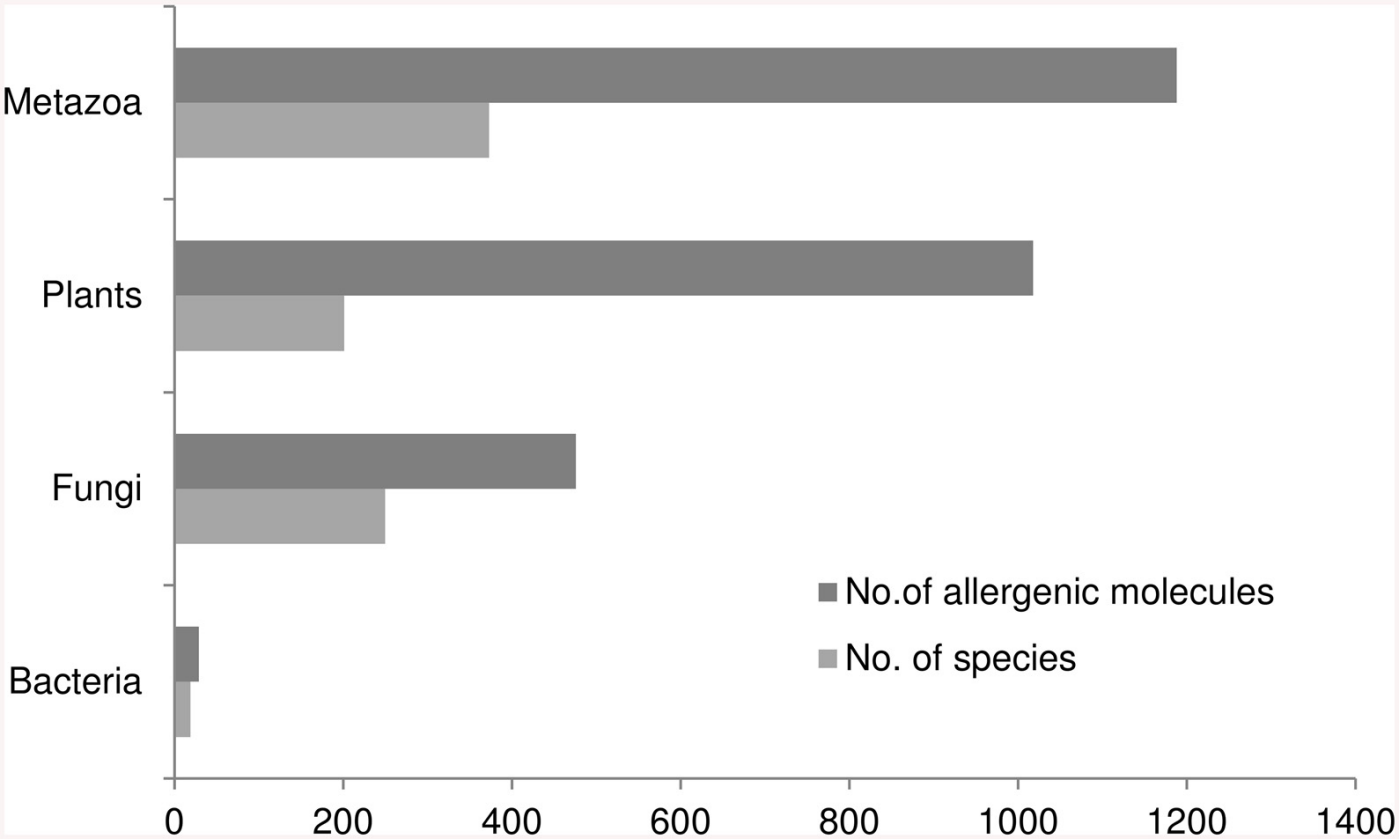
The distribution of protein molecule entries (dark gray bars) listed in the Allergome database across different species (light gray bars) by taxonomic grouping (Bacteria, Fungi, Plants and Metazoan).

The distribution across different taxonomic groups of the 2712 allergenic molecules listed in the Allergome database for which IgE-binding antigen is reported suggests that allergenic/IgE-binding protein molecules are moderately represented in fungal (17.5% of all Allergome entries) genomes and poorly represented in bacterial genomes (~1%) (Fig 2) and are consistent with the previously published studies [47,48]. A large proportion of these molecules are represented in plant (~38%) and metazoan genomes (44%). Interestingly, these large proportions of allergens in metazoa and plants (1018 plant and 1188 metazoan allergens) are encoded in genomes of a restricted number of species (201 plant and 373 metazoan species, respectively).

### Representation of allergens/IgE-antigens in Pfam

**Fig 3:**
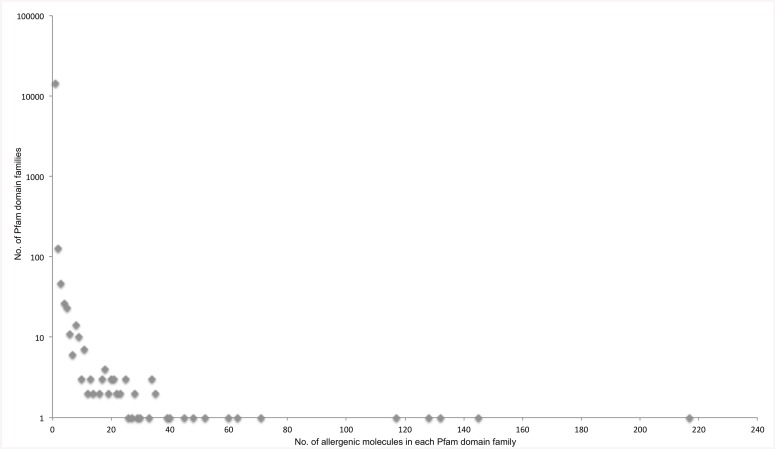
Distribution of allergenic molecules retrieved from Allergome database across Pfam domain families. Number of Pfam domain families with no allergenic members have also been represented. The Y-axis is scaled logarithmically (base 10), however true values are represented.

**Fig 4:**
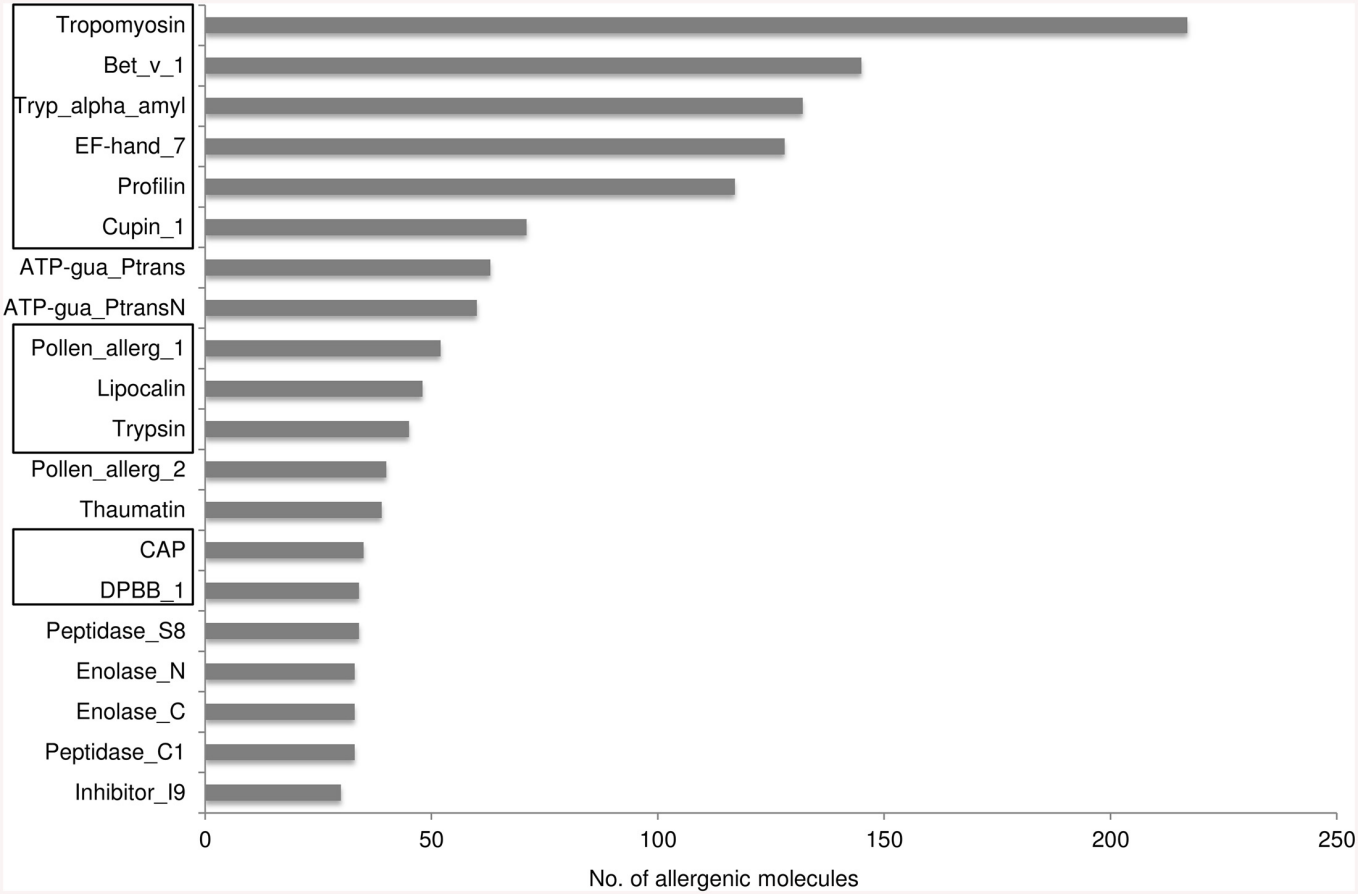
Pfam domain families that are highly populated with allergenic protein sequences. Protein domain families considered for this analysis are highlighted in the box based on the families presented in the article co-authored by Fitzsimmons and Dunne [21].

The 2712 unique UniProt protein sequence entries corresponding to protein molecules listed in the Allergome database are distributed in 331 Pfam protein domain families. Protein domain families with allergenic entries are relatively few and account for merely 2.2% of total 14831 protein domain families specified in Pfam database (Fig 3). There are 128 protein families that are comprised of only one allergenic protein member. A total of 1389 out of 2712 allergenic molecules (~51%) are members of just 20 protein domain families (Fig 4). Among these families, Tropomyosin (Pfam accession: PF00261) constitutes 217 allergenic molecules (~8% of all allergens).

### Average length of a linear epitope

A large proportion of known epitopic regions in proteins have been determined by employing a 'peptide fragment approach'. The IEDB database provides a curated list of these epitopic regions along with a limited set of epitopes that are determined by analysing structures of antigen-antibody complexes. The majority (58%) of linear epitopic regions in the IEDB database demonstrated to bind to IgE/IgG4 are within peptides of 10 and 15 amino acid residues length. This is consistent with the average length of an epitope determined based on analysing structures of antigen and antibody complexes [49].

### Categorization of allergenic protein domain families/superfamilies

We have chosen 10 protein domain families/superfamilies that have been referred to in the ‘Opinion article’ co-authored by Fitzsimmons and Dunne [21] for our analysis. These families/superfamilies, encompassing nearly 40% of all allergenic proteins, differ in terms of their distribution across different taxonomic groups as well as experimental details available, and can be categorized into three groups (A, B, C) depending on: (i) whether protein families/superfamilies include parasite protein representatives as well as known allergens; and (ii) whether IgE-binding immunoassays have/have not been performed on parasite proteins (Table 2).

**Table 2:** Categorization of 10 protein domain families into groups.

	Homologs in metazoan parasite	IgE activity tested	Protein domain families
**Group A**	✓	✓	Tropomyosin, EF Hand and CAP
**Group B**	✓	✗	Profilin, Lipocalin, Trypsin-like serine protease, Cupin, Bet v 1 and Expansin
**Group C**	✗	✗	Prolamin

#### Group A

Protein families categorized in this group are populated with members of allergenic and parasite proteins and hence homologies have already been established. In this group, positive IgE-binding activity has been demonstrated for the parasite proteins, indicating clear-cut evidence for the allergenic potential of proteins. It includes Tropomyosin, EF Hand and CAP (**C**ysteine-rich secretory proteins, **A**ntigen 5, and **P**athogenesis-related 1 proteins) protein domain families.

#### Group B

Protein families belonging to this group represent protein members from allergenic and parasitic sources; however, none of the parasite proteins have yet been tested for antibody binding activity. We have employed protein sequence and structure based computational methods to identify putative epitopic-like regions for the families categorized in this group, arriving at a comprehensive list of putative allergenic proteins encoded in genomes of parasites that can be prioritized for carrying out IgE-binding immunoassays. Group B includes Profilin, Lipocalin and Trypsin-like serine protease, Cupin, Bet v 1 and Expansin protein families.

#### Group C

This group contains only the prolamin protein domain family, which has no representation as yet in metazoan parasitic organisms and hence no IgE-binding activity has been tested so far. This group may represent those parasite proteins where only a small fragment potentially shares similarity to the known allergen.

### Homologs of allergenic proteins in parasite protein sequence dataset

#### Groups A and B

871 unique UniProt protein sequence entries corresponding to worm (Platyhelminth and nematode) and mite sources are associated with the same Pfam domain families as all allergenic proteins in group A (EF hand, CAP and Tropomyosin) and three of those in group B (Profilin, Lipocalin and Trypsin-like serine protease). These constitute close homologs of the allergenic proteins. Protein members in families categorised in group A and trypsin-like serine proteases in group B are distributed across prokaryotic and eukaryotic organisms. However, Profilin and Lipocalin families categorised in Group B show representation only from eukaryotic and eukaryotic metazoan organisms, respectively.

For detecting homologs of allergenic proteins in the 3 remaining group B families (Bet v 1, Cupin and Expansin), we used parasite protein annotations specified by Gene3D. Allergenic proteins with known PDB structures belonging to families in group B are categorised in the following CATH homologous superfamilies: a) Bet v 1: 3.30.530.20 b) Cupin: 2.60.120.10 c) Expansin proteins (DPBB_1, Pollen_allerg_1): 2.40.40.10 and 2.60.40.760, respectively. For Bet v 1 and Cupin domain families, we detected 58 and 130 parasite proteins that are indexed with CATH accession numbers 3.30.530.20 and 2.60.120.10 respectively and hence are in same homologous superfamily as allergenic proteins. These parasite proteins are considered as close homologs of allergenic proteins. One of the Bet v 1 -like proteins from *S*. *mansoni* was chosen for expression and testing in human IgE binding studies (see below).

Prior to this study, databases for the expansin domain family contained no parasite proteins, indicating that no member from a parasite had previously been found.

No parasite proteins with CATH accession number 2.40.40.10 and 2.60.40.760 were detected, which indicates that no parasite protein member has previously been found to correspond to expansin proteins from allergenic sources and thus we could not find ‘close homologs’ of allergens in expansin domain family (with same CATH accession number). However, we could find distant homologs’ in metazoan parasites proteome for the same domain family (at protein fold level).

Therefore, for establishing distant homology, 1387 parasite proteins that share the same topology/fold as the allergenic expansin proteins were collected. Homologs of allergenic proteins in parasites (belonging to Group A and B) have been listed in S1 Table.

Known 3D structures/reliable 3D models of parasite proteins and corresponding allergenic proteins in group B were compared using structure superposition. For further analysis, we restricted the list of parasite proteins that share structural similarity with allergenic proteins with a TM score of ≥0.5. This list constitutes 9 Bet v 1 and 2 Cupin parasite proteins that share homology with allergenic proteins. We also find 15 parasite proteins that are expected to share the same topology as expansin proteins, thereby representing distant homologs. A list of these parasite proteins is provided in S2 Table.

#### Group C

No ‘true’ parasite protein sharing homology or even overall topology/fold with Prolamin could be detected. However, Gene3D does categorize a protein from *S*. *mansoni* (Uniprot accession: G4VGF5) with the prolamin protein (Uniprot accession: P01085, PDB accession 1HSS) from *Triticum aestivum* (Wheat) in CATH superfamily 1.10.110.10. The algorithm employed by Gene3D aligns only a small fragment of the worm protein (from residue 406–436) with the wheat protein and is not considered statistically significant.

We also detected a protein (UniProt accession: Q1M2M1) encoded in genome of *Glycyphagus domesticus* (House itch mite), which shares significant homology with prolamin protein Mal d 3 (UniProt accession: Q9M5X7) from *Malus domestica* (Apple). However, with careful inspection, we learnt that the mite protein shares a sequence identity of 100% with lipid transfer protein (UniProt accession: M8BYH8) from *Aegilops tauschii* (Tausch's goatgrass). This finding suggests a possible sequence artifact with regard to the mite protein and hence, is not considered as a true prolamin protein from the mite. At this stage, the absence of parasitic homologs for prolamin can partly be attributed to paucity of data.

### Prediction of putative IgE binding regions in homologs of allergenic proteins in parasite proteins

#### Sequence based approach

We next predicted putative IgE/IgG4-binding regions for various family members in parasite proteins. The Allergome database details those proteins encoded in genomes from various sources for which IgE binding activity has already been studied. We have predicted putative IgE binding regions for parasite proteins (EF hand, 4 and tropomyosin, 151) by comparing them with fragments of allergens for which IgE binding activity has been ascertained experimentally (S3 Table).

Using this approach, we are able to identify parasite proteins belonging to group A and B that share significant sequence similarity with known IgE binding fragments, comprising a total of 177 metazoan parasite proteins (EF hand: 48, Tropomyosin: 48, Profilin: 12, Lipocalin: 23 and CAP: 46). Results corresponding to the predicted IgE binding regions are presented in S4 Table. However, this sequence based approach did not yield any significant results for Bet v 1, Cupin and Expansin protein members (Group B).

#### Structure based approach

A total of 344 3D structural motifs that are known to bind IgE/IgG4 were searched in a dataset (3D known structures/3D homology models) of 26 parasite proteins belonging to Bet v1, Cupin and Expansin families (sharing TM score ≥0.5 with allergenic proteins) using the BC-search program (S2 Table). BC-search is a novel program, which employs a Binet-Cauchy kernel to decipher protein fragment structural similarities at a local level. The extent of shape similarity is measured on the basis of statistically significant BC scores, which are length independent even for shorter fragments, where measure of RMSD could be misleading. BC-search performed 211,852 structural fragment comparisons along all the parasite proteins and reported a score for each comparison. Specific regions in all 26 parasite proteins displayed significant shape similarity (BC score ≥0.8) to the structural motifs of known antibody binding fragments.

Information on 3D structural motifs that are known to bind IgE/IgG4 with their structural equivalents on 26 parasite proteins and their corresponding topological positions has been provided in S5 Table.

Epitope regions for Class B allergens showing significant matches (calculated by BC search program) with parasite proteins has been provided in S6 Table.

### Examples of putative IgE targets in metazoan parasites

Illustrative examples of newly detected metazoan parasite protein which are potential IgE target are discussed in the following section.

#### Group A (EF-hand)

**Fig 5:**
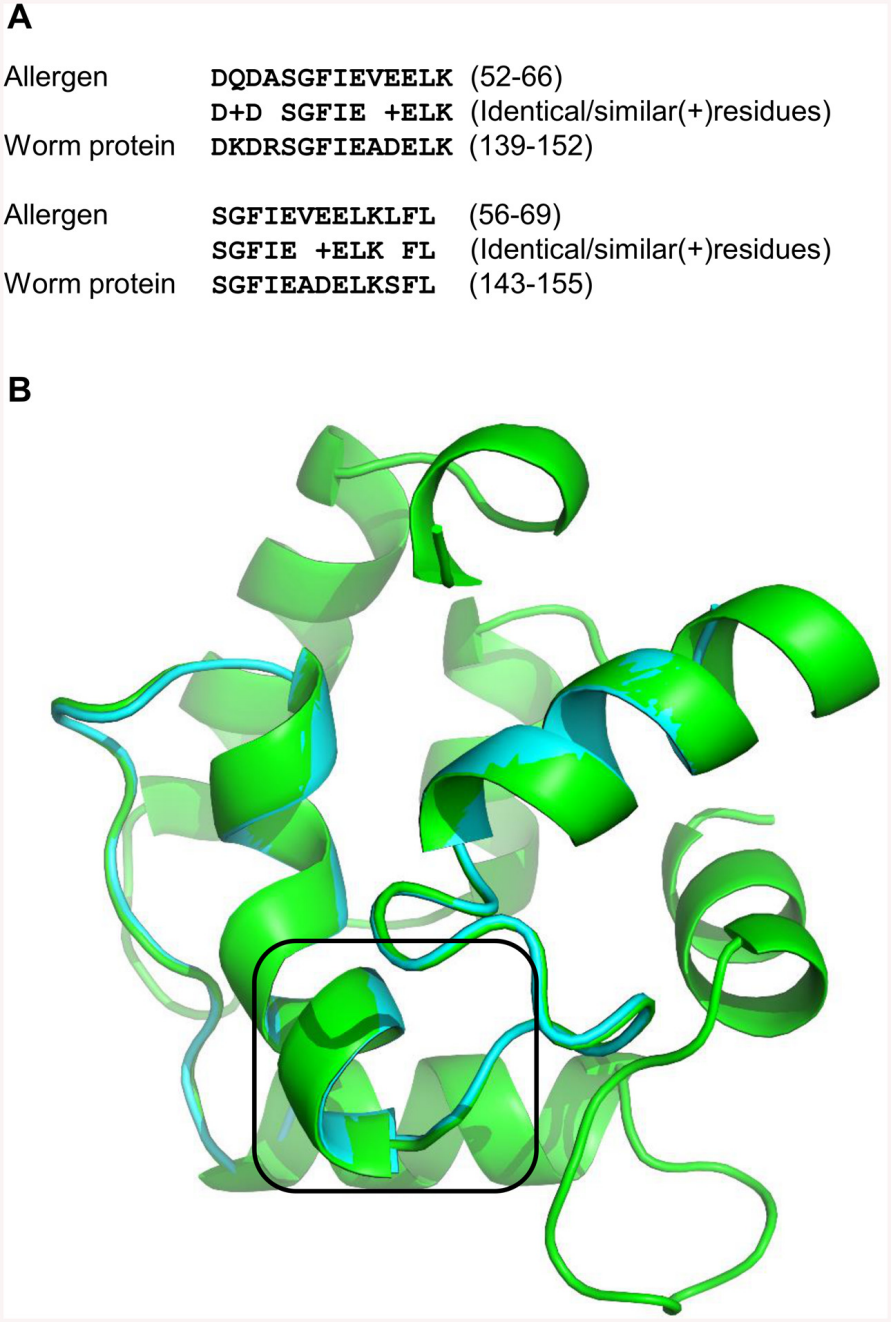
**A. Sequence alignment of the epitopic region from Atlantic salmon (*Salmo salar*) allergenic parvalbumin-like 1 protein (UniProt accession: B5DH17) and predicted epitopic-like region from worm *Schistosoma japonicum* (UniProt accession: Q5C262).** B. Superposition of the 3D structural model of salmon parvalbumin-like 1 protein (colored in green) and EF hand domain of the *Schistosoma japonicum* protein (in cyan). Epitope (allergen) and predicted epitopic-like regions (parasite protein) are depicted in the box.

EF-hand domain regions from a putative calcium ion binding protein from *Schistosoma japonicum* (UniProt accession: Q5C262) exhibit similarity with allergenic Parvalbumin protein sequence ‘Sal s 1’ from Atlantic salmon (UniProt accession: B5DH17). Epitopes on the allergenic protein have been mapped using peptide microarray immunoassays [50]. While parasitic and allergenic proteins share only 13% sequence identity, epitopic regions (from residues 52–66 and 56–69) from the allergenic protein share sequence identity of 71% and 76% respectively with the predicted epitopic like regions from the worm protein (from residues 139–152 and 143–155) (Fig 5A). Reliable 3D structural models for the allergenic protein and EF hand domain region from the worm protein were modelled using parvalbumin from *Gallus gallus* (PDB accession: 3FS7) as a template. Root mean square deviation values for the generated models (allergen and worm EF hand domain) with respect to the template proteins are 0.13Å and 0.21Å respectively. Superposition of 3D models of both the proteins is depicted in Fig 5B.

#### Group B (Profilin)

**Fig 6:**
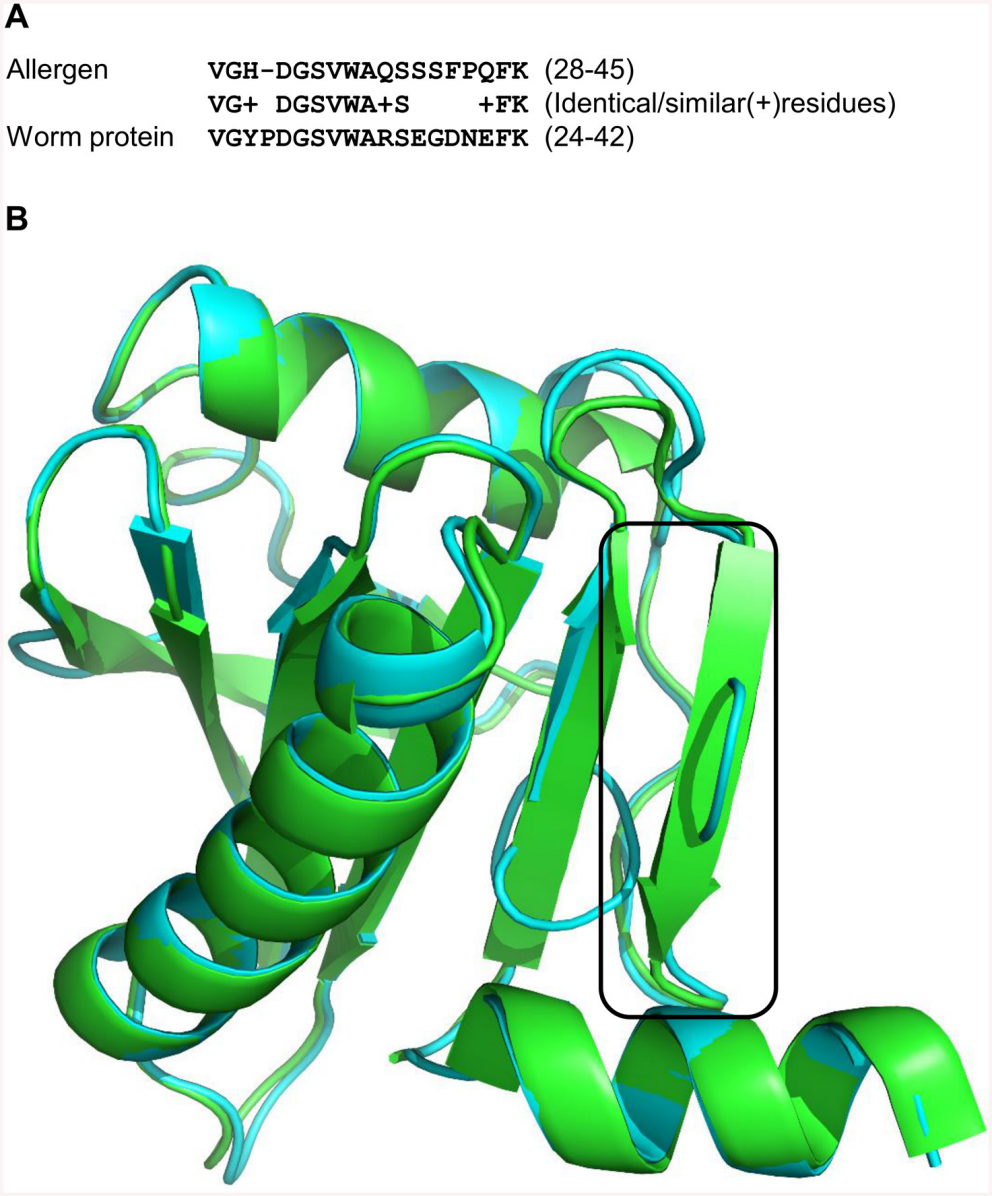
**A. Sequence alignment of the epitopic region from profilin protein (allergenic) from *Betula pendula* (European white birch) (UniProt accession: P25816) and predicted epitopic-like region from the worm *Ascaris lumbricoides* (UniProt accession: F1LGV9).** B. Superposition of the 3D structure of plant allergenic protein (PDB accession: 1CQA) (colored in green) and the 3D structural model of profilin protein from the worm *Ascaris lumbricoides* (in cyan). Epitope (allergen) and predicted epitopic-like regions (parasite protein) are depicted in the box.

We predict a potential IgE target protein from the nematode worm *Ascaris lumbricoides* (UniProt accession: F1LGV9) belonging to Profilin domain family. The worm protein shares homology with pollen allergen from plant *Betula pendula* (European white birch). The allergen also has been annotated as a Profilin protein according to the domain definition provided by Pfam. The two proteins share 27% sequence identity. However, the predicted epitopic-like regions of the *Ascaris* protein (residues 24–42) show significant sequence identity of 61% (similarity of 76%) with known epitopic regions of plant allergen (from residue 28 to 45) [51] (e-value 6e-05), as shown by the sequence alignment of these regions in Fig 6A. A 3D model of the worm protein and known 3D structure of the allergenic protein (PDB accession: 1CQA) share gross structural similarity with Cα RMSD of 0.65Å (Fig 6B).

Performing a similar analysis, we have also predicted potential IgE targets from *S*. *mansoni* (UniProt accession: G4VTE3) and *Brugia malayi* (UniProt accession: A8Q2M4, corresponding to Lipocalin family) for which IgE activity has been tested in the laboratory. We have confirmed prevalent IgE activity for a Profilin protein from *S*. *mansoni* (using human sera) and a lipocalin protein from *B*. *malayi* (in vivo studies) when compared with a control protein.

#### Group B (Expansin)

**Fig 7:**
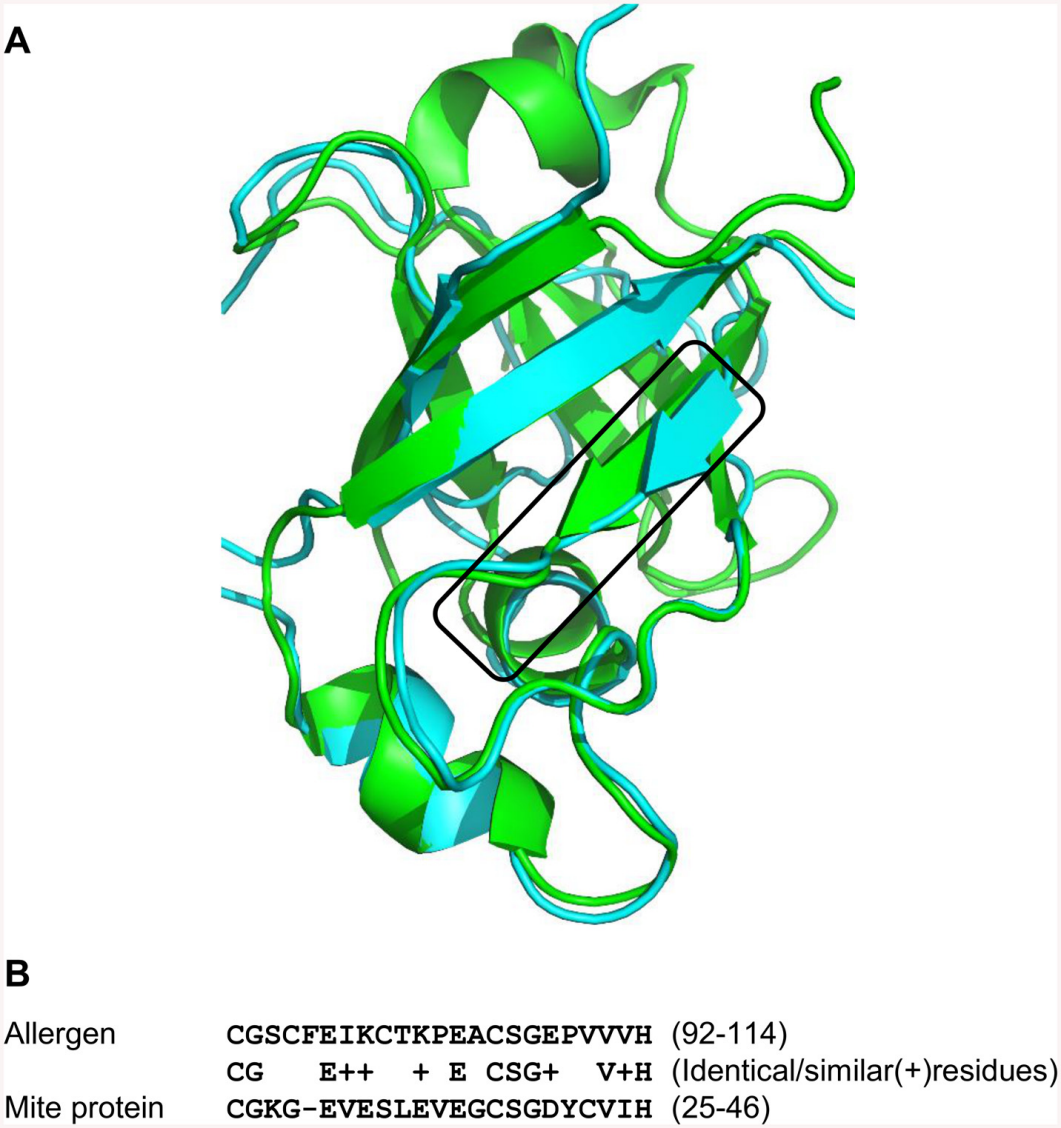
**A. Superposition of the 3D structure of timothy grass Phl p 1 (PDB accession: 1N10) (in green) and the 3D structural model of mite protein (in cyan). Epitope (allergen) and predicted epitopic-like regions (parasite protein) are depicted in the box.** B. Sequence alignment of the epitopic region from *Phleum pratense* ‘Phl p 1’, a major timothy grass pollen allergen (UniProt accession: P43213) and predicted epitopic-like region from Mite group 2 allergen ‘Pso o 2’ protein (UniProt accession: Q965E2) from *Psoroptes ovis*.

A further example from group B is the Mite group 2 allergen ‘Pso o 2’ protein (UniProt accession: Q965E2) [52] from *Psoroptes ovis* (Sheep scab mite), which shares the same topology as an allergenic protein from the expansin family and thus shares distant homology. Overall, this mite protein has only 11.4% sequence identity with ‘Phl p 1’, a major timothy grass pollen (UniProt accession: P43213; PDB accession: 1N10). However, epitopes from the plant allergen protein are known and have been identified using IgE binding immunoassays [53]. Superposition of a 3D model of the mite and known 3D structure of the allergen is shown in Fig 7A (TM score ~0.6).

This analysis establishes a clear similarity between epitope and predicted epitopic-like regions from allergen and mite protein. These regions share sequence identity of 40% and similarity of 63% (Fig 7B), thus establishing structural similarity between epitopes of a plant allergen and an expansin-like protein encoded in the genome of metazoan parasite. These regions show BC score (measure of structure similarity) of 0.996 with RMSD and p-value of 0.36Å and 2.06658e-08 respectively.

### Confirmation of predicted IgE/IgG4 binding by a *S*. *mansoni* Bet v 1-like protein

The plant-specific Bet v1 domain family represents the second large family which is highly populated with allergens (see Fig 4). However, no Bet v1-like IgE target proteins have been reported in metazoan parasites so far.

We tested the potential of Bet v 1-like protein (SmBv1L) from *S*. *mansoni* predicted by our computational analysis to bind IgE and IgG4 in plasma from individuals infected with this parasite. The Bet v 1-like protein from *S*. *mansoni* (Uniprot accession: G4VE06) and the plant birch pollen allergen (Uniprot accession: P15494, PDB accession: 1BV1) are categorised in the same homologous superfamily (CATH accession: 3.30.530.20) as described by the CATH database. SmBv1L was cloned and expressed as a recombinant protein in *E*.*coli*. Insoluble recombinant protein was extracted from inclusion bodies, purified and refolded on column to give soluble antigen. The presence of a single protein species of approximately 18 kDa, as predicted *in silico*, was confirmed by a Coomassie blue-stained SDS-PAGE gel.

**Fig 8:**
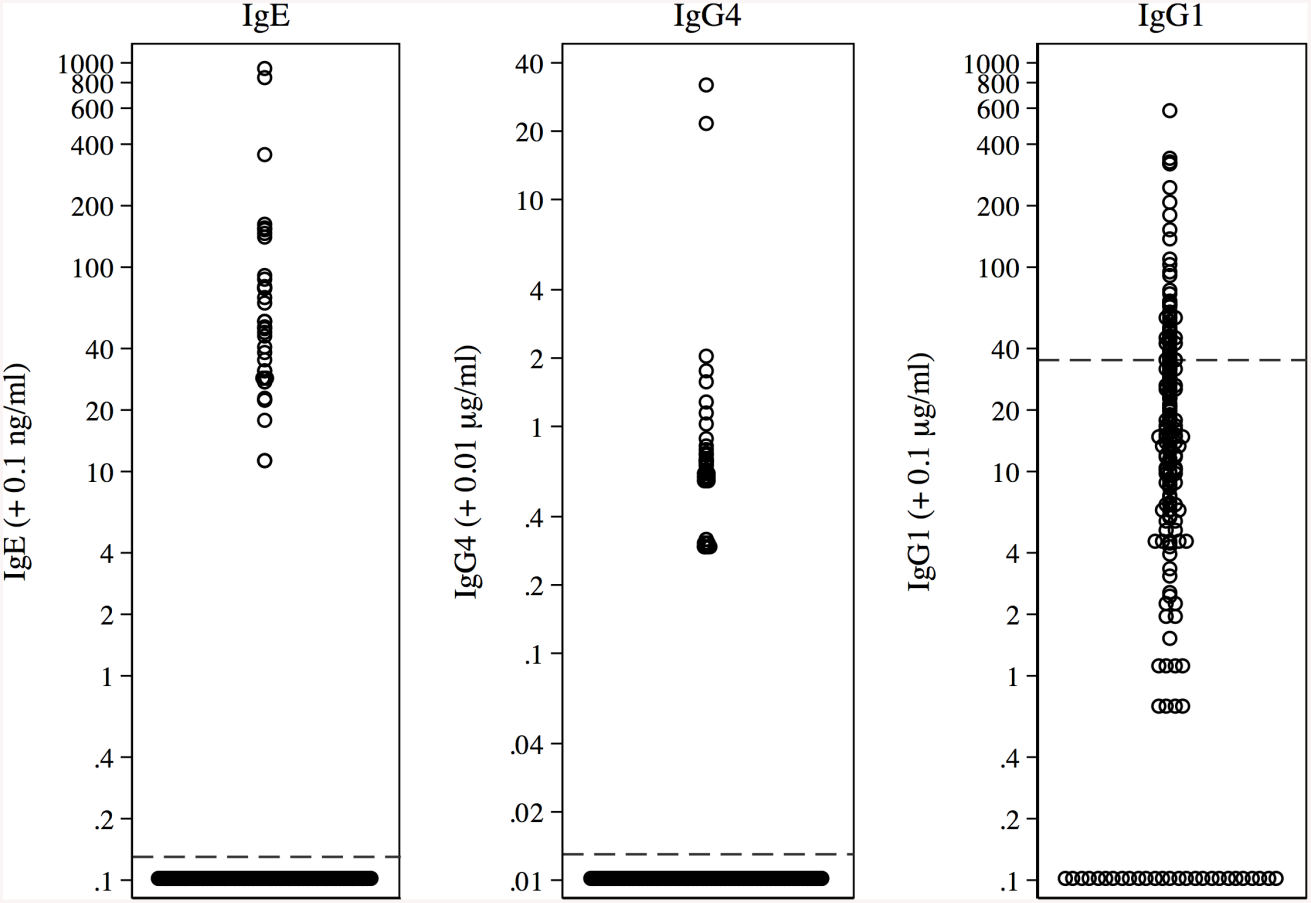
Magnitudes of specific IgE, IgG4 and IgG1 responses to *S*. *mansoni* Bet v 1-like protein, SmBv1L, in a population of 222 individuals infected with *S*. *mansoni*, dotted lines indicate threshold of magnitude for a response. Data were normalized for expression on a log scale by the addition of constants so as to include zero values.

**Fig 9:**
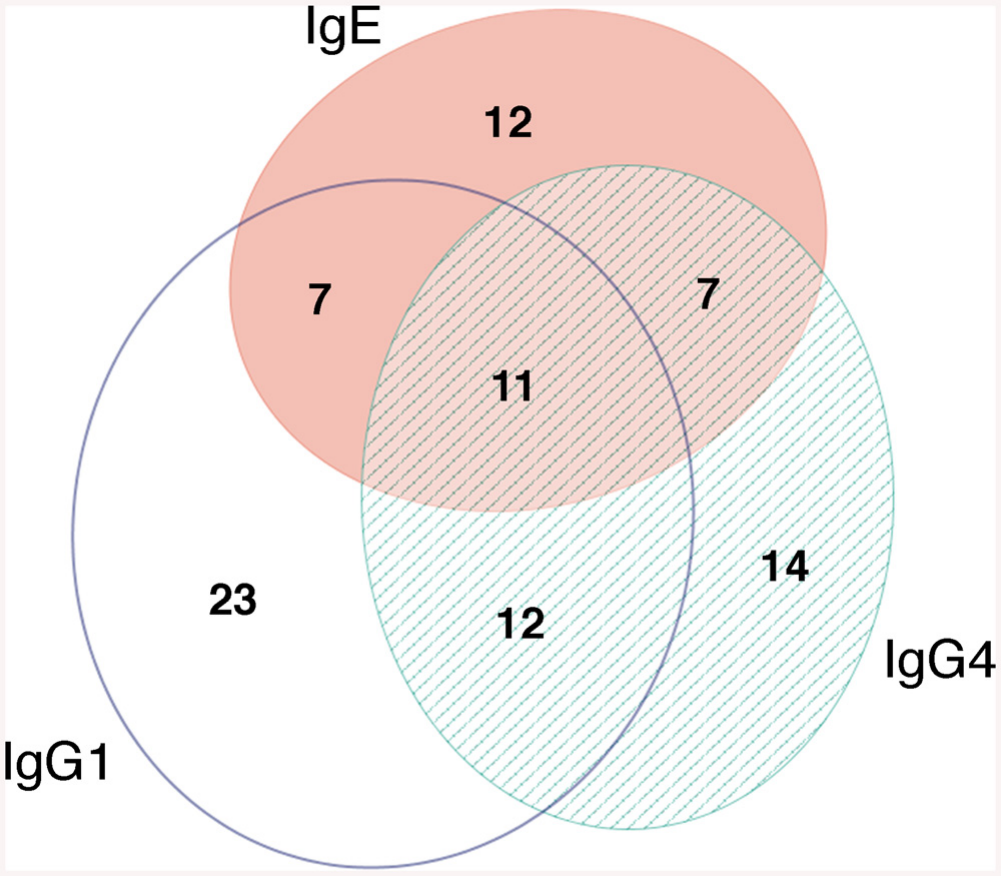
Venn diagram showing the distribution of Ab isotype responses to SmBv1L within IgG1, IgG4 and IgE responders in a population of 222 individuals endemically infected with *S*. *mansoni*.

Antibody responses against SmBv1L were measured in a population of 222 *S*. *mansoni*-infected individuals from a parasite endemic area in Uganda. Levels of antigen specific responses were measured against IgG1, IgE and IgG4 (Fig 8). SmBv1L was found to be antigenic with 38.7% of the population responding with IgG1, IgG4 or IgE. Antibody responses against SmBv1L with IgG1, IgG4 and IgE were found in 23.9% (53), 19.8% (44) and 16.7% (37) of the population respectively. The geometric mean magnitudes of responder antibody levels were as follows IgG1; 70.2 μg/ml (95% CI 57.9, 85.1 μg/ml), IgG4; 0.7 μg/ml (95% CI 0.5, 0.9 μg/ml) and IgE; 56.6 ng/ml (95% CI 40.4, 79.5 ng/ml). As shown by Fig 9, individuals were capable of producing responses against IgG1, IgG4 or IgE alone or any combination of the three. There were no significant differences between the overlaps of any combination of the three antibody isotypes.

## Discussion

The IgE-mediated immunological effector mechanisms that cause allergy are similar to those associated with immunity to metazoan parasites. IgE responses elicited in cases of helminth infections can be considered to be a mammalian adaptation evolved to provide protection against helminth and arthropod parasites. It is hypothesised that in the absence of helminth infection, allergenic proteins can be mistakenly targeted by the same arm of the immune system which originally evolved to combat parasitic infection, resulting in an unregulated (sometimes lethal) allergic response.

Metazoan parasites are more closely related to their mammalian host than lower taxonomic groups such as bacteria, and viruses. It is remarkable that IgE responses are more strongly targeted to eukaryotic and not prokaryotic proteins. However, based on analyses of three animal allergen protein domain families (EF hand, tropomyosin and casein), Jenkins and co-workers have inferred that allergenic proteins are sufficiently diverged (<62% sequence identity) from the corresponding homologous proteins from the human host [54]. Interestingly, the number of protein domain families that include the allergenic molecule is also limited and a large proportion of these allergenic molecules are encoded in the genomes of a limited set of plant and metazoan species. Also, a limited set of proteins from parasitic organisms will be in contact with host machinery to evoke any immune response, which would also depend on the protein expression profile of parasite at different life stages [55]. These observations indicate that a narrow range of molecules from metazoan parasites and environmental allergens are responsible for eliciting immune/antigenic response [21].

Previously, IgE binding activity has been studied for certain metazoan parasite proteins corresponding to domain families (such as EF hand, Tropomyosin and CAP) that also represent a major proportion of protein sequences from allergenic sources giving an indication about possible sequence and structure similarity with the allergenic proteins [21]. However, even for these families, consolidated information regarding sequence and structure similarity with parasite proteins has not been demonstrated. To validate and reinforce our underlying hypothesis, it becomes crucial to have a systematic and comprehensive catalogue of closely related parasite proteins and allergens that can be tested as targets for IgE.

A novel systematic approach involving sensitive remote homology detection methods has been applied to identify and document proteins encoded in genomes of various metazoan parasites, which share homology (sequence and structure based) with the IgE/IgG4 binding allergenic molecules. Structure-based methods proved to be helpful in establishing remote homology as well as inferring topological similarity of epitopic regions between allergenic and parasite proteins where sequence based methods were not effective. We categorised predicted target parasite proteins into three groups based on (a) if they share homology with allergenic protein (b) if immunoassays have been performed to test IgE/IgG4 activity. Using various computational approaches, we also identified putative epitopic-like regions in parasite proteins that might have potential for IgE binding.

Tropomyosin is the largest family that represents the maximum number of known allergens from worms and arthropods (Fig 4). However, the second most prevalent domain family is the Bet v1 family, of which no potential IgE targets have been identified in metazoan parasites. We tested the predictions made for a Bet v 1-like protein (SmBv1L) from *S*. *mansoni* using *in vitro* IgE/IgG4 binding studies. SmBv1L was the target for IgE responses in the inhabitants of a Ugandan schistosomiasis mansoni endemic area, confirming the prediction of the bioinformatics pipeline. The geometric mean IgE level of 56.6 ng/ml (23.6 IU/ml), was comparable to specific IgE responses to common known allergens [56]. Additionally, IgG4 responses against SmBv1L were observed, indicating that IgG4 mediated regulatory responses, as seen against other *S*. *mansoni* allergen-like proteins and in allergy, were induced by SmBv1L [27–29]. To our knowledge, SmBv1L represents the first confirmed example of a Bet v 1-like protein in metazoan parasites that is targeted by IgE, and indeed one of the few known examples of a Bet v 1-like antigen from a non-plant organism. The identification of a Bet v 1-like molecule in *S*. *mansoni* that is phylogenetically disparate from other known allergens of the same family highlights the relevance of the proposed hypothesis and sensitivity of the methodology applied to detect and establish such distant relationships that could be supported by immunological assays performed *in vitro*.

In our recent work, as a control, we also tested Adenylate kinase (AK), protein 14-3-3 and Ubiquitin, antigens that were shown to be prevalent in the human life cycle stages of *S*. *mansoni*, in our pipeline for IgE binding structural features and epitopes. These antigens were not predicted to bind IgE by our pipeline, a finding supported by our previously published data [57]. This demonstrated that whilst AK, 14-3-3 and Ubiquitin were antigenic, generating prevalent IgG1 responses in the infected cohort, there was no strong evidence for IgE binding by these antigens with IgE responses being of both low magnitude and prevalence.

By using our methodology, we have established such relationships for 9 out of 10 protein domain families that contain highest numbers of known allergens. Homology for allergenic prolamin protein members was not established with parasite proteins. Gene3D did detect some similarity between the CATH prolamin superfamily and a domain in a *Schistosoma mansoni* protein, but the region detected was too small to be statistically significant. This small region does however map to known epitope regions in prolamin found in wheat. It is tempting to speculate that the region being detected corresponds to the fragment that, by happenchance, shares the IgE interaction motif. Additionally, the lack of detection could be attributed to many factors such as scarcity of information regarding IgE–binding epitopes, incomplete genomes of parasitic organisms and unavailability of 3D structural information regarding allergens and parasite proteins. However, the possibility that this ‘plant storage family’ is indeed absent from metazoan parasites cannot be ruled out completely. Nonetheless, our analyses lend support to our hypothesis by drawing clear comparisons between allergenic and metazoan parasite proteins in rest of the 9 protein domain families.

The lack of details for the molecular mechanisms involved in immunity and allergic states and lack of exclusive descriptions of allergenic molecules such as, for example, the presence of conserved motif(s) limit our understanding of immune dysfunction in allergic disease. It is still unclear what molecular mechanisms lead these small epitopes to elicit an IgE-dominated allergic response. By demonstrating that we can detect similarities between epitopes of multicellular parasite proteins and those from allergy-inducing environmental and food proteins, we lay the foundation for studying predicted IgE binding targets, to aid our understanding of the underlying mechanisms involved in both immunity against parasites and in an allergenic response. This understanding is of paramount importance since the IgE response would not have evolved to cause allergy. Defining allergen-like molecules in parasites and understanding their link to the unregulated IgE response will therefore facilitate the discovery and design of molecules for future immunotherapy in allergic conditions.

### Ethics statement

The Ugandan National Council of Science and Technology provided ethical clearance for the human serology studies. Consent forms were translated in the local language and informed written consent was obtained from all adults and from the parents/legal guardians of all children under 15.

## Supporting Information

S1 Table Homologs of allergenic proteins belonging to families EF hand, CAP, Tropomysoin, Profilin, Lipocalin, Trypsin-like serine protease, Bet v 1, Cupin and Expansin in metazoan parasites. (XLS)

S2 Table List of homologs of allergenic protein in parasites that share TM score of > = 0.5 with the allergenic protein. (XLSX)

S3 Table List of putative IgE binding regions in proteins (belonging to EF hand and tropomyosin domain families) mentioned in the Allergome database. (XLS)

S4 Table List of putative IgE binding regions in proteins (belonging to EF hand, Tropomyosin, CAP, Profilin and Lipocalin domain families) in parasite proteins. (XLS)

S5 Table Representation of topology of epitopic regions on allergen and corresponding structural motifs on parasite proteins for Group B families/superfamilies (Bet v1, Cupin and Expansin). (PDF)

S6 Table Epitope regions for Class B allergens showing significant matches (calculated by BC search program) with equivalent regions in parasite proteins. BC scores, p-value and RMSD have been calculated for the structural alignments of epitopes and their equivalent motifs on parasite proteins.
(XLSX)
