# Supplementary material for: Comparisons of Allergenic and Metazoan Parasite Proteins: Allergy the Price of Immunity
Source: PLoS Comput Biol. 2015 Oct 29;11(10):e1004546. doi: 10.1371/journal.pcbi.1004546 (PMC4626114; doi:10.1371/journal.pcbi.1004546)
Supplement: S5 Table — (PDF) [file pcbi.1004546.s005.pdf]

Supplementary Information SI 5: Representation of topology of epitopic regions on allergen and corresponding structural motifs on parasite proteins for Group B families/superfamilies (Bet v1, Cupin and Expansin).

Homologs of allergens (Column 2,3) in parasites (Column 4,5) categorized in Group B. Epitopic fragments in allergens (column 6) and their structural equivalent fragments in parasite proteins (column 7) have been shown.  BC score, p-value and RMSD are calculated for structural alignment of epitopic fragments and corresponding regions in parasite proteins.

| Group B families | Allergen (Uniprot accession code) | Allergen molecule | Eukaryotic metazoan parasite protein | Organism | allergen_start_end_residue | worm_start_end_residue | BC score | p-value | RMSD (calculated for small fragment epitope of the allergen and structural equivalent of the parasite protein | Structural alignment of allergen molecule (in cyan) and parasite protein (in green). The epitope of the allergen and structural equivalent of the parasite protein are colored in pink and yellow respectively. |
|---|---|---|---|---|---|---|---|---|---|---|
| Bet v1 | P43179 | Major pollen allergen Bet v 1-F/I | A8PG94 | Brugia malayi | 136-156 | 205-225 | 0.916143 | 6.28732e-08 | 0.907802 | 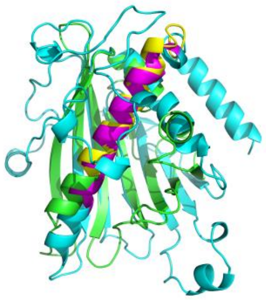 |
| Bet v1 | P43179 | Major pollen allergen Bet v 1-F/I | A8Q7R1 | Brugia malayi | 136-156 | 204-224 | 0.919016 | 6.03997e-08 | 0.888846 | 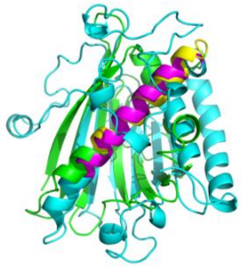 |

| | | | | | | | | | | |
|---|---|---|---|---|---|---|---|---|---|---|
| Bet v1 | P15494 | Major pollen allergen Bet v 1-A | A8QE91 | Brugia malayi | 25-34 | 324-333 | 0.967007 | 3.08941e-08 | 0.676474 | 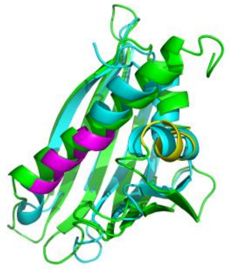 |
| Bet v1 | P15494 | Major pollen allergen Bet v 1-A | C4QT89 | Schistosoma mansoni | 49-58 | 97-106 | 0.890527 | 8.99237e-08 | 1.516553 | 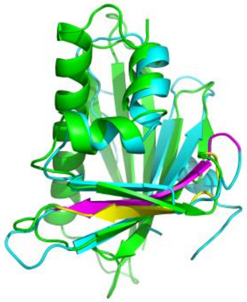 |
| Bet v1 | P15494 | Major pollen allergen Bet v 1-A | E5S942 | Trichinella spiralis | 139-152 | 269-282 | 0.985771 | 2.37702e-08 | 0.324278 | 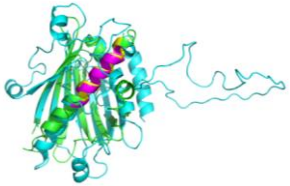 |

| | | | | | | | | | | |
|---|---|---|---|---|---|---|---|---|---|---|
| Bet v1 | P15494 | Major pollen allergen Bet v 1-A | F1KTC4 | Ascaris suum | 25-34 | 23-32 | 0.958435 | 3.48244e-08 | 0.735116 | 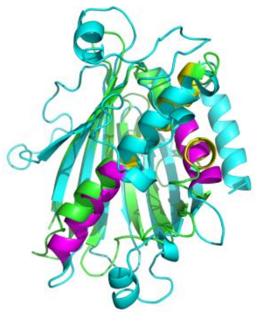 |
| | | | | | 139-152 | 204-217 | 0.994626 | 2.10045e-08 | 0.235949 | |
| Bet v1 | P15494 | Major pollen allergen Bet v 1-A | Q5DCR9 | Schistosoma japonicum | 131-153 | 221-243 | 0.985054 | 2.40097e-08 | 0.347401 | 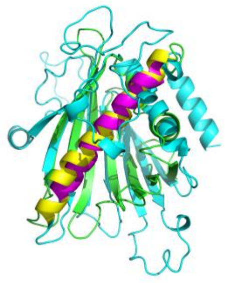 |
| Bet v1 | P15494 | Major pollen allergen Bet v 1-A | G4V7D7 | Schistosoma mansoni | 25-34 | 320-329 | 0.964278 | 3.20945e-08 | 0.642115 | 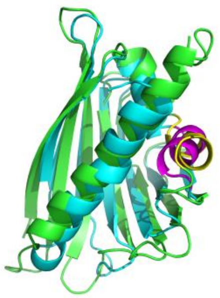 |

| | | | | | | | | | | |
|---|---|---|---|---|---|---|---|---|---|---|
| Bet v1 | P15494 | Major pollen allergen Bet v 1-A | G4V7D8 | Schistosoma mansoni | 25-34 | 233-242 | 0.917058 | 6.20744e-08 | 0.857349 | 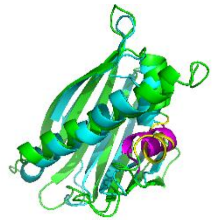 |
| Cupin | P43238 | Allergen Ara h 1 | A8PRG7 | Brugia malayi | 439-453 | 59-73 | 0.858167 | 1.41320e-07 | 1.562960 | 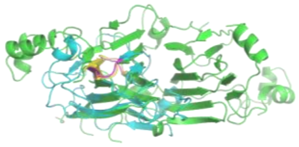 |
| Cupin | P43238 | Allergen Ara h 1 | A8NVE6 | Brugia malayi | 502-516 | 48-62 | 0.939144 | 4.55951e-08 | 0.775005 | 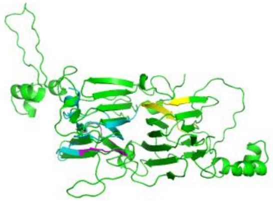 |

| Group B families | Allergen (Uniprot accession code) | Allergen molecule | Eukaryotic metazoan parasite protein | Organism | Allergen_ start_end_ residue | Parasite_pr otein_start_ end_residue | BC score | p-value | RMSD (calculated for small fragment epitope of the allergen and structural equivalent of the parasite protein ) | Structural alignment of allergen molecule (in cyan) and parasite protein (in green). The epitope of the allergen and structural equivalent of the parasite protein are colored in pink and yellow respectively. |
|---|---|---|---|---|---|---|---|---|---|---|
| Expansin -like | P43213 | Pollen allergen Phl p 1 | Q965E2 | Sheep scab mite | 95-113 | 27-45 | 0.995790 | 2.0665 8e-08 | 0.362591 | 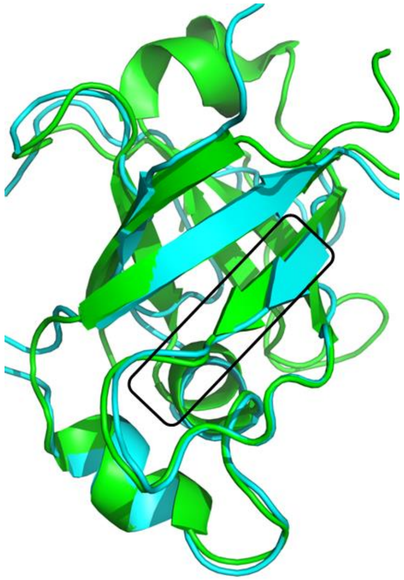 *Explained in the main text in detail.* |

| | | | | | | | | | | |
|---|---|---|---|---|---|---|---|---|---|---|
| Expansin-like | Q947S4 | Acidic Cyn d 1 isoallergen isoform 4 | G4VRS4 | Schistosoma mansoni | 190-199 | 431-440 | 0.910484 | 6.80453e-08 | 0.590549 | 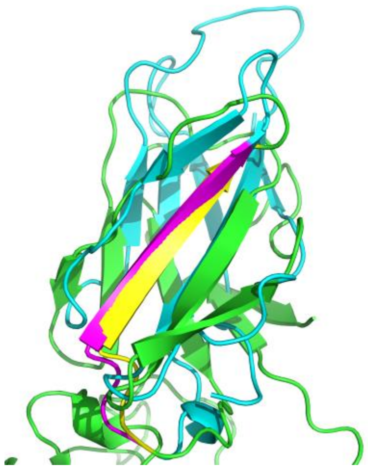 |
| Expansin-like | O04701 | Major pollen allergen Cyn d 1 | A8NU08 | Brugia malayi | 231-240 | 359-368 | 0.944553 | 4.22768e-08 | 0.957344 | 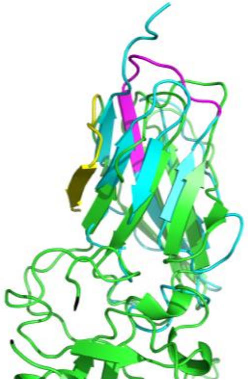 |

| | | | | | | | | | | |
|---|---|---|---|---|---|---|---|---|---|---|
| Expansin-like | O04701 | Major pollen allergen Cyn d 1 | A8NVE8 | Brugia malayi | 191-200 | 834-843 | 0.958768 | 3.46624e-08 | 0.623835 | 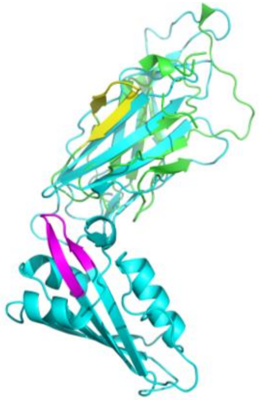 |
| Expansin-like | P43216 | Major pollen allergen Hol l 1 | A8P222 | Brugia malayi | 183-203 | 1605-1625 | 0.918215 | 6.10798e-08 | 1.534991 | 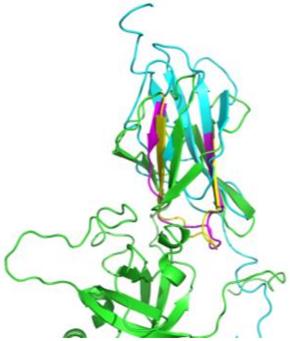 |

| | | | | | | | | | | |
|---|---|---|---|---|---|---|---|---|---|---|
| Expansin-like | O04701 | Major pollen allergen Cyn d 1 | A8PZX3 | Brugia malayi | 231-240 | 438-447 | 0.891808 | 8.83296e-08 | 1.920115 | 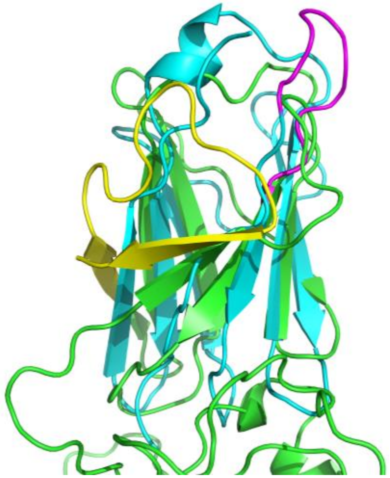 |
| Expansin-like | P43213 | Pollen allergen Phl p 1 | A8Q0N7 | Brugia malayi | 118-128 | 484-494 | 0.967306 | 3.07654e-08 | 1.343974 | 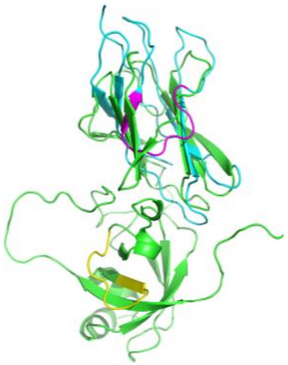 |

| | | | | | | | | | | |
|---|---|---|---|---|---|---|---|---|---|---|
| Expansin -like | O04701 | Major pollen allergen Cyn d 1 | A8Q7G0 | Brugia malayi | 191-200 | 196-205 | 0.942151 | 4.37198e-08 | 4.858769 | 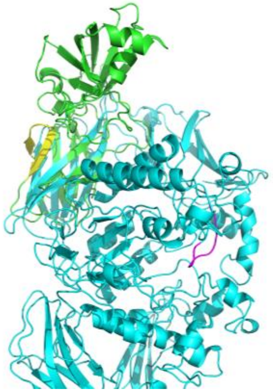 |
| Expansin -like | O04701 | Major pollen allergen Cyn d 1 | A8QFI4 | Brugia malayi | 231-240 | 1837-1846 | 0.884845 | 9.73532e-08 | 1.103646 | 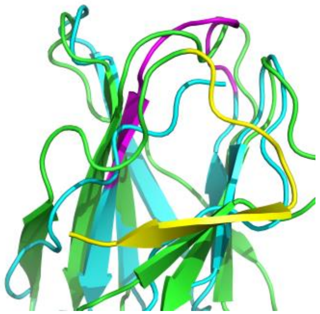 |

| | | | | | | | | | | |
|---|---|---|---|---|---|---|---|---|---|---|
| Expansin -like | O04701 | Major pollen allergen Cyn d 1 | A8QGM5 | Brugia malayi | 71-80 | 564-573 | 0.900504 | 7.8224 6e-08 | 0.866005 | 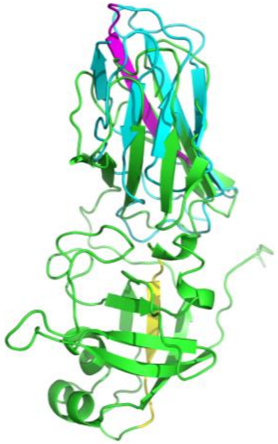 |
| Expansin -like | O04701 | Major pollen allergen Cyn d 1 | E5T7N2 | Trichinella spiralis | 226-235 | 97-106 | 0.876628 | 1.0919 5e-07 | 2.164496 | 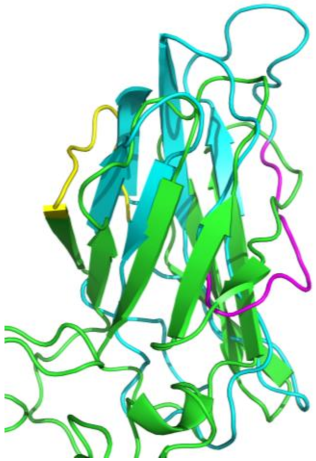 |

| | | | | | | | | | | |
|---|---|---|---|---|---|---|---|---|---|---|
| Expansin-like | O04701 | Major pollen allergen Cyn d 1 | G4V6J3 | Schistosoma mansoni | 191-200 | 856-865 | 0.967571 | 3.06515e-08 | 0.573590 | 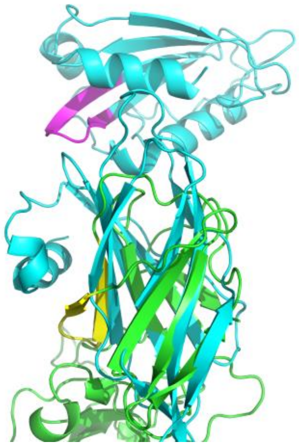 |
| Expansin-like | O04701 | Major pollen allergen Cyn d 1 | G4V6K2 | Schistosoma mansoni | 101-110 | 253-262 | 0.953752 | 3.71784e-08 | 1.890765 | 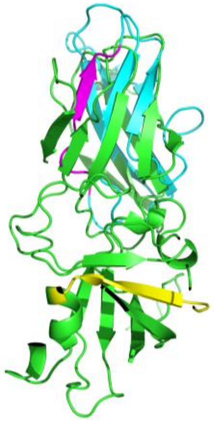 |

| | | | | | | | | | | |
|---|---|---|---|---|---|---|---|---|---|---|
| Expansin -like | O04701 | Major pollen allergen Cyn d 1 | Q5DA33 | Schistosoma japonicum | 70-79 | 65-74 | 0.881513 | 1.01991e-07 | 0.697432 | 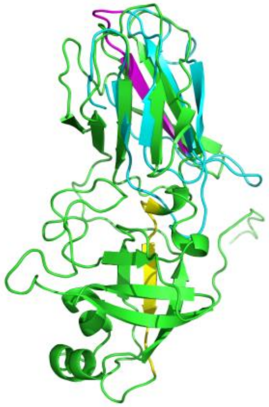 |
| Expansin -like | P43213 | Pollen allergen Phl p 1 | Q5DHP4 | Schistosoma japonicum | 241-262 | 52-73 | 0.920083 | 5.95065e-08 | 3.699460 | 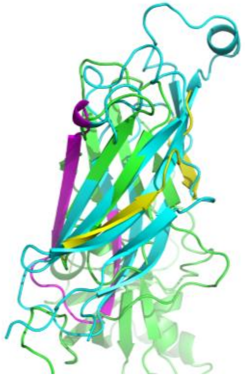 |
